# Study of Partial Oxidation of Methane by Ni/Al_2_O_3_ Catalyst: Effect of Support Oxides of Mg, Mo, Ti and Y as Promoters

**DOI:** 10.3390/molecules25215029

**Published:** 2020-10-29

**Authors:** Ahmed A. Ibrahim, Wasim U. Khan, Fahad Al-Mubaddel, Ahmed S. Al-Fatesh, Samsudeen O. Kasim, Sofiu L. Mahmud, Ateyah A. Al-Zahrani, M. Rafiq H. Siddiqui, Anis H. Fakeeha

**Affiliations:** 1 Chemical Engineering Department, College of Engineering, P.O. Box 800, Riyadh 11421, Saudi Arabia; wasimkhan@gmail.com (W.U.K.); falmubaddel@ksu.edu.sa (F.A.-M.); sofkolajide2@gmail.com (S.O.K.); mahmudsofiu@gmail.com (S.L.M.); aazz@ksu.edu.sa (A.A.A.-Z.); anishf@ksu.edu.sa (A.H.F.); 2 King Abdullah City for Atomic & Renewable Energy: Energy Research & Innovation Center (ERIC) in Riyadh, Riyadh 11451, Saudi Arabia; 3 Chemistry Department, King Saud University, P.O. Box 2455, Riyadh 11451, Saudi Arabia; rafiqs@ksu.edu.sa

**Keywords:** Al_2_O_3_, CH_4_, partial oxidation, support promoters, synthesis gas, Ni catalyst

## Abstract

Catalysts of 10% Ni, supported on promoted alumina, were used to accomplish the partial oxidation of methane. The alumina support was doped with oxides of Mo, Mg, Ti and Y. An incipient wetness impregnation technique was used to synthesize the catalysts. The physicochemical properties of the catalysts were described by XRD, H_2_-TPR (temperature programmed reduction), BET, TGA, CO_2_-TPD (temperature-programmed desorption) and Raman. The characterization results denoted that Ni has a strong interaction with the support. The TGA investigation of spent catalysts displayed the anticoking enhancement of the promoters. The impact of the support promoters on the catalyst stability, methane conversion and H_2_ yield was inspected. Stability tests were done for 460 min. The H_2_ yields were 76 and 60% and the CH_4_ conversions were 67 and 92%, respectively, over Ni/Al_2_O_3_+Mg, when the reaction temperatures were 550 and 650 °C, respectively. The performance of the present work was compared to relevant findings in the literature.

## 1. Introduction

The depletion of fossil fuel resources despite their growing demand and utilization has posed prominent concerns. Thus, researchers focused on the transformation of natural gases into invaluable chemical compounds [1,2]. Natural gas, which is mainly composed of CH_4_ is often converted into useful products such as synthesis gas (H_2_ and CO), which is versatile and beneficial in producing chemicals like methanol and ethane [3,4,5]. At present, many technologies exist for CH_4_ reforming to generating synthesis gas. Steam reforming which is used on a large scale has H_2_/CO of 3, while dry reforming which has attracted the attention of many investigators due to the environmental aspects, generates synthesis gas of H_2_/CO of 1 [6,7,8,9,10]. Currently, partial oxidation of CH_4_ (POM) is seen to be a substitute method for both steam and dry reforming in synthesis gas production. POM draws the consideration due to its benefits, such as mild exothermicity, high efficiency, and giving an appropriate H_2_/CO ratio of 2 and, therefore, it can prevent several disadvantages of the endothermic reaction of steam or dry reforming of CH_4_ [11,12,13]. Catalytic methods used in the production of synthesis gas via POM can be categorized into two main metal-based groups: transition catalysts and noble catalysts [14,15,16,17,18]. Noble metals such as Rh, Pt and Pd are costly which render their utilization an economic issue, even though they are active and stable for the conversion of CH_4_ [19]. It is obligatory to seek appropriate substitutes which are not expensive. The transition metals like Ni, Co and Fe are the feasible choices [20]. Ni catalysts have been broadly examined due to their cheap cost and comparatively higher activity in the POM. Nevertheless, Ni-based catalysts are hampered by rapid catalyst deactivation as a result of Ni sintering [21,22] and phase changing of the supported constituents during the reaction [23]. Recently, researchers have been engaged in exploring the possibilities of optimizing Ni-based catalysts which are branded for a cheap price and high activity. The optimization technique stipulates wide dispersion of metal particles in the catalyst by selecting proper support, which limits the sintering and aggregation of the active species [22]. It is evident that the performance of Ni catalysts relies on the support [24]. Moreover, to alter metal-supported interactions, several metal oxides with high surface area and thermal stability like Al_2_O_3_, MgO, TiO_2_ and the like have been used to minimize deactivation [25]. The mixture of Al_2_O_3_ and MgO form spinel of MgAl_2_O_4_ which is characterized by high resistance to coke depositions through stronger interactions with nickel nanoparticles [26]. Furthermore, the MgO component has the same crystal structure as NiO. The NiO−MgO solid solution develops a strong ionic bond between metal phase and support that reduces coke formation [27]. Recent studies of Ni-based catalysts have been performed with alumina or silica supports [28]. Zhang et al. reported that Ni supported on aluminate displays better activity and stability than on other supports [29]. Jalali et al. investigated Ni-based nanocatalysts employing various kinds of support for the production of synthesis gas through combined dry and partial reforming of CH_4_ [30]. The results of activity and stability performance denoted that the Ni catalyst supported on NiAl_2_O_4_ displayed a 2% rise in activity. In the study of Alvarez-Galvan et al., the influence of support type on POM to produce synthesis gas, using wet impregnation and solid-state reaction, was presented [31]. They found alumina support gave the best performance on both preparation methods. This was credited to the high presence of Ni on the alumina surface. Diaz and Assaf studied CO_2_ reforming of CH_4_ by promoting the alumina support with basic metal oxide and they found that the modified support diminished the carbon deposition and retarded the deactivation [32]. Claude et al. reported the improvement of the catalytic performances and lifespan of Ni/γ-Al_2_O_3_ catalysts doped with species like Mo for steam reforming of toluene [33]. Mo-doped Ni/γ-Al_2_O_3_ catalysts exhibited the high reforming activity of toluene and low quantities of carbon formation. The highest performance was registered at 650 °C. Zhong-yu et al. [34] investigated the impact of Cu and Mo components of γ-Al_2_O_3_-supported Ni catalysts on hydro deoxygenation of fatty acid methyl esters. The promoters demonstrated better catalytic competence than Ni/γ-Al_2_O_3_ catalysts. Mo in the catalysts had two states: Mo^4+^ and Mo^6+^. Shah et al. examined the dry reforming of CH_4_ employing an Ni-based catalyst and TiO_2_-Al_2_O_3_ combined as support [35]. The results displayed that doping of Al_2_O_3_ with TiO_2_ boosted the catalytic activity and stability. Li-jun et al. [36] studied C_3_H_8_ catalytic combustion employing Ag-Mn/γ-Al_2_O_3_-TiO_2_ catalysts. Results showed that composite support promoted the dispersion of Ag and Mn elements on the surface of the catalyst and created a strong interaction between the active metals, in order to augment the relative amount of reactive O_2_ species and enhance the reducibility of catalysts, which upgraded the catalytic activity of C_3_H_8_ combustion. Zhang et al. [37] tested the catalytic performance of NiMo support on Al_2_O_3_-TiO_2_ for HDS of 4, 6-dimethyldibenzothiophene. The composite support evidenced great specific surface area, high thermal stability and slight pore size distributions. The addition of TiO_2_ could affect the interactions between metal and support, therefore, improved the reduction of active metals. The catalytic results revealed that the conversion over the catalysts slowly increased as the Ti/Al molar ratio increased to 0.4. Abdollahifar et al. used an Ni-Co/Al_2_O_3_–MgO nanocatalyst for H_2_ production by dry reforming of CH_4_ [38]. The nanosynthesized Ni-Co/Al_2_O_3_–MgO displayed the suitability of the active phases in the presence of the combined support. Alibi et al. [39]; reported the benefit of using Ni and Ni-Co support on a mixture of Mg and alumina-supported catalysts with various concentrations of Mg and Al for dry reforming of methane. Their results displayed a remarkable enhancement of conversion, selectivity, and resistance to carbon formation, particularly when the Mg/Al ratio was bigger than unity. These effects were due to the improvement of active metal reduction, metal support and metal−metal interactions. Khoja et al. [40] also investigated the influence of the combined support of Mg and Alumina for the same reaction, employing a cold plasma dielectric barrier discharge reactor. They found that the addition of MgO to Ni/Al_2_O_3_ helped to increase the basicity of the support and enhanced the interaction of Ni and Al_2_O_3,_ which affected the reducibility and stability. Jung et al. [41] studied steam reforming of CH_4_, employing a series of Ni-MgO-Al_2_O_3_ catalysts for fuel cells. They tested MgO loadings of 3 to 15 wt%. Their results displayed the highest conversion of methane and resistance against K poisoning when a 10 wt.% MgO support was used. The superb performance of reforming is related to the large interaction of Ni and alumina support when MgO is coprecipitated with the Ni-Al_2_O_3_. The improved interaction of the Ni with MgO−Al_2_O_3_ support protected the Ni against K poisoning. Özdemir and Öksüzömer examined the performance of Al_2_O_3_, MgO and MgAl_2_O_4_ supports on 10% Ni catalyst in the partial oxidation of methane [41,42]. The catalyst supported by the combination of Mg and Al showed the highest activity and selectivity. High conversion of CH_4_ and constant H_2_/CO for 20 h was reported. Yttria (Y_2_O_3_) is extensively used as a sintering additive to strengthen Al_2_O_3_ [43]. Y_2_O_3_ is reported as an effective additive in preparing ceramics to increase mechanical properties by minimizing interfacial reactions, increasing oxidation resistance, consolidating grain boundaries and regulating grain size [44]. Y_2_O_3_ has been reported to form NiYO_3_ compound with Ni, causing the alteration of coke and coke location in the autothermal reforming of methane [45]. Hongbo et al. [46] performed steam reforming of ethanol at a low temperature for hydrogen production using Ni/Y_2_O_3_-Al_2_O_3_ catalysts. They used Y_2_O_3_- Al_2_O_3_ with different mole ratios. The result of composite support had a suitable synergistic effect between the active constituent and support, and NiO could be reduced easily and hence the composite support catalysts displayed high activity for ethanol steam reforming. Santos et al. [45] studied the influence of yttrium oxide addition to Ni/α-Al_2_O_3_ catalysts in autothermal reforming of CH_4_. The Y_2_O_3_-Al_2_O_3_ supported catalysts offered high performance. The upgraded stability of catalysts resulted from the decrease of coke on the surface of Ni. A similar reaction process was performed by Sun et al. [47] who investigated the impacts of Y_2_O_3_-doping to Ni/γ-Al_2_O_3_ catalysts during the autothermal reforming of CH_4_ to H_2_ and CO. The results of different loadings of Y_2_O_3_ (5%, 8%, 10%) showed substantial enhancement in performance. The Ni supported on Y_2_O_3_+γ-Al_2_O_3_ had greater NiO reducibility, minor Ni particle size, wider Ni dispersion than those of the pristine Ni/γ-Al_2_O_3_ catalysts. Therefore, the mixture support repressed the sintering of Ni, transformed the type of coke and reduced the content of coke on the catalysts.

The purpose of this study is to work out the best textural promoter of Al_2_O_3_ support on Ni catalyst to achieve high activity and stability, while minimizing the carbon formation during the POM process. The influence of promoters like oxides of Y, Ti, Mg, and Mo on the activity, stability, and coke formation of promoted γ-Al_2_O_3_ supported on Ni catalysts were surveyed. Numerous characterization techniques were used to better understand the catalytic performance.

## 2. Results and Discussion

The textural properties of Ni-Al-x (x = 0, Mo, Ti, Y, and Mg) were evaluated using the N_2_ adsorption−desorption isotherms. Supplementary Figure S1 displays the N_2_ adsorption−desorption isotherms of the fresh catalysts calcined at 650 °C. According to the IUPAC classification, the catalysts display a type IV isotherm with a hysteresis loop of the H3-type, resulting from capillary condensation and evaporation at elevated P/P_0_ [48]. The rise in the amount of N_2_ adsorbed at high P/P_0_ in the isotherm curves is due to the phenomenon of capillary condensation within the sample pores. The sorption isotherms of all the catalysts are alike, designating that there is almost no change in the pore structure of the support when modified with the metal oxides. Table 1 depicts the BET surface area, average pore diameter, and pore volume.

**Figure 1:**
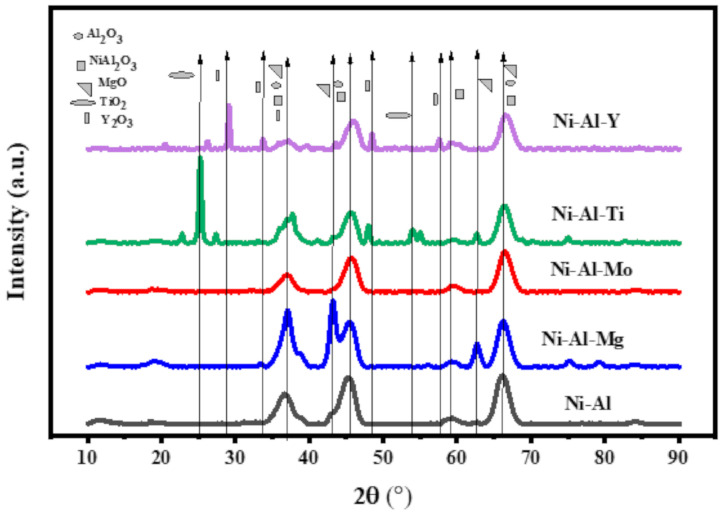
The XRD profiles of Ni-Al-x (x = 0, Mo, Ti, Y, and Mg) catalysts.

Figure 1 displays the XRD profiles of fresh Ni-Al-x (x = 0, Mo, Ti, Y, and Mg) catalysts. The samples showed the XRD peaks of γ-Al_2_O_3_ at 2θ = 37.2, 45.8 and 66.4° (JCPDS 86-1410). The peaks for NiO overlapped those of γ-Al_2_O_3_ and appeared at 2θ = 37.2, 45.8 and 66.4° (JCPDS 47-1049), with the reflections of 111, 200, 220, respectively, suggesting that these Ni-containing phases were highly dispersed on the surface of the supports. The modification of the alumina support with Mo did not have any influence on the characteristic peaks depicting the uniform scattering Mo in the matrix of Ni-Al. On the other hand, the addition of 10% of Mg, Ti and Y oxides maintained the characteristic peaks of the Ni/γ-Al_2_O_3_. However, some additional peaks appeared related to the particular metal oxide. For instance, the characteristic peaks of MgO occurred at 2θ = 43.3° and 63.1°, the peaks of TiO_2_ appeared at 2θ = 25.1° and 54.3°, while Y_2_O_3_ appeared at 2θ = 29.0°, 48.4° and 58.1° (JCPDS 083-0927).

**Figure 2:**
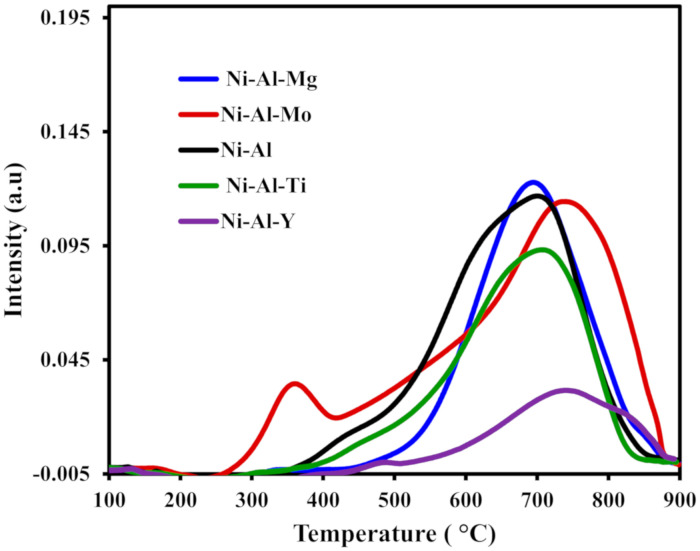
Temperature programmed reduction (TPR) profiles of the fresh Ni-Al-x (x = 0, Mo, Ti, Y, and Mg) catalysts.

Figure 2 shows the H_2_-TPR (temperature programmed reduction) curves of the fresh catalysts: Ni-Al-x (x = 0, Mo, Ti, Y, and Mg). No clear reduction peak was detected in the range of 200−400 °C for all the catalysts except the catalyst supported on Mo-modified alumina, signifying the nonappearance of dissociated or free NiO in the prepared catalysts. However, for the case of the Mo-modified catalyst, there was an additional reduction peak at around 300−440 °C, which denoted the weak interaction of dissociated or free NiO with Al_2_O_3_-Mo support. Virtually all the catalysts were characterized by two peaks. The peak that appeared before 500 °C, with varying intensities, could be ascribed to moderate interaction between NiO and the Al_2_O_3_. The second broad reduction peak appeared within the range of 700–880 °C. This was assigned to NiAl_2_O_4,_ which resulted from the strong interaction of Al_2_O_3_ with NiO [49]. The addition of promoters to the support did not enhance the reducibility of the catalysts according to the TPR profiles.

### Catalytic Performance

**Figure 3:**
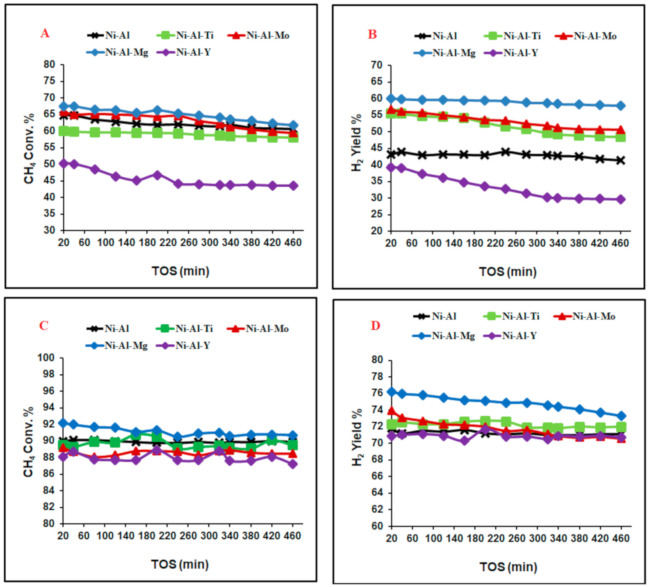
(**A**) CH_4_ conversions reaction temperature 550 °C; (**B**) hydrogen yield reaction temperature 550 °C; (**C)** CH_4_ conversions reaction temperature 650 °C; (**D**) hydrogen yield reaction temperature 650 °C; as a function of time-on-stream over the Ni catalysts. (mass of catalyst, 0.1 g; CH_4_:O_2_ = 2:1, 1 atom; and flow rate, 32.5 mL*/*min.).

The activities of all five catalysts: Ni-Al-x (x= Mo, Ti, Y, and Mg), and Ni-Al were investigated in partial oxidation of CH_4_ over the reduced catalysts under the same reaction conditions at 550 and 650 °C for approximately 8 h. The outcomes of the experiments are shown in Figure 3. Figure 3A depicts the CH_4_ conversion as a function of time on stream (TOS). On the one hand, no substantial reduction in conversion was identified in the activity of Ni-Al-Ti catalyst, which suggests the stability of the catalyst. The catalyst had 60% initial CH_4_ conversion and nearly maintained that value. On the other hand, Ni-Al-Mg, Ni-A-Mo and Ni-Al_2_O_3_ catalysts gave the highest initial CH_4_ conversions of about 68.0, 67.7, 67.7% with reductions of 5.7, 8.1, 8.1%, respectively, over the time on stream. Ni-Al-Y catalyst gave the lowest initial conversion of 50% and a 6.6% reduction. Figure 3B displays the H_2_ yield versus time on stream. Ni-Al-Mg catalyst gave the highest initial H_2_ yield of about 60%, while the Ni-Al-Y catalyst presented the lowest initial H_2_ yield of about 39.2%. Figure 3C shows the CH_4_ conversion versus time on stream setting the temperature at 650 °C. Higher CH_4_ conversions were observed as a result of the increase in the reaction temperature. No sizable reduction in conversion was observed for all catalysts, suggesting the existence of good stability in their TOS. Ni-Al-Mg catalyst gave the highest CH_4_ conversion of about 92%, whereas the Ni-Al-Y catalyst provided the least conversion of about 88%. Figure 3D shows the H_2_ yield profiles against TOS. The stability of the catalysts was quite good since the drop in the yield of H_2_ was less than 3% in all cases over the TOS. The Ni-Al-Mg catalyst gave the greatest H_2_ yield of about 76% while the Ni-Al-Y catalyst had the least H_2_ yield of about 71%. The promoting alumina with Mg, Mo and Ti enhanced the performance of the catalysts, while the Y promoter lowered the performance. These characteristics of promoters were obvious at lower reaction temperatures. Supplementary Figures S2 and S3 display the CO yield versus time on stream at 550 and 650 °C reaction temperatures, respectively.

Table 2 compares the efficiency of the catalysts used in this work to the partial oxidation results obtained by other investigators. It is evident that the work performed in this manuscript is worth sharing with other investigators in the field.

**Figure 4:**
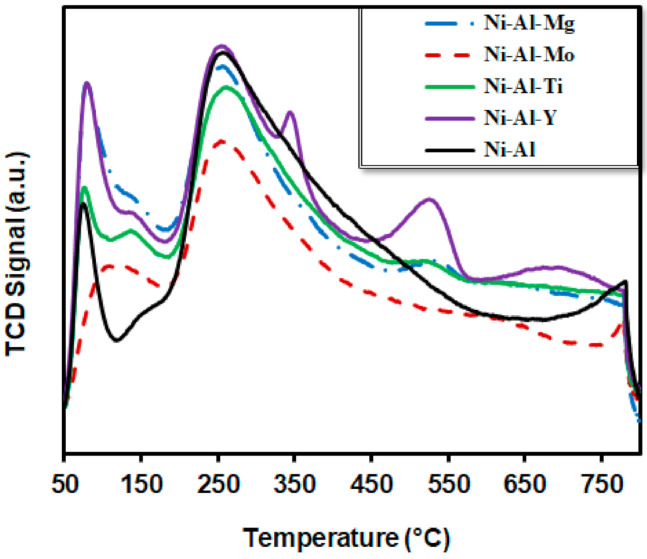
Represents the CO_2_-temperature-programmed desorption (TPD) profiles of the Ni-Al-x (x = 0, Mo, Ti, Y, and Mg) catalysts.

The strength type of basic sites can be categorized by the temperature of the related desorption peaks: the ranges of 50–200 °C, 200–400 °C, 400–650 °C, above 650 °C, are commonly ascribed to the weak, intermediate, strong and very strong, respectively. Figure 4 depicts the CO_2_ temperature-programmed desorption, which evaluates the basicity of Ni-based catalysts on the basic sites at different temperatures.

For the Ni-Al catalyst, there were peaks at 72, 245, and at 772 °C, attributable to the weak, intermediate, and very strong basic sites, respectively. For the Ni-Al-Mo catalyst, a similar pattern of peaks was observed at 102, 245 and 775 °C, indicating that the addition of Mo did not alter the basicity. For the Ni-Al-Mg catalyst, there were three peaks. The first two peaks were similar to the unmodified alumina supported catalyst; however, the third peak appeared at 522 °C, denoting strong but not very strong basicity. For the Ni-Al-Ti catalyst, there were four peaks at 73, 124, 248 and 510 °C. Two peaks were in the weak basicity region, while the remaining two peaks were similar to that of Mg-modified support, showing intermediate and strong basicity. However, when Y was promoted with the alumina, six peaks were observed in its profile. Two of them were in the weak basicity region, where a peak was observed at 77 °C with a shoulder desorption peak of low intensity at 133 °C. Another two peaks were in the intermediate basic region, at 244 and 340 °C. The remaining two peaks appeared at 515 and 703 °C and described as strong and very strong peaks. The Y-modified supported catalyst exhibited relatively higher intensity and wider CO_2_ desorption area peaks, suggesting more basicity than unmodified alumina support.

**Figure 5:**
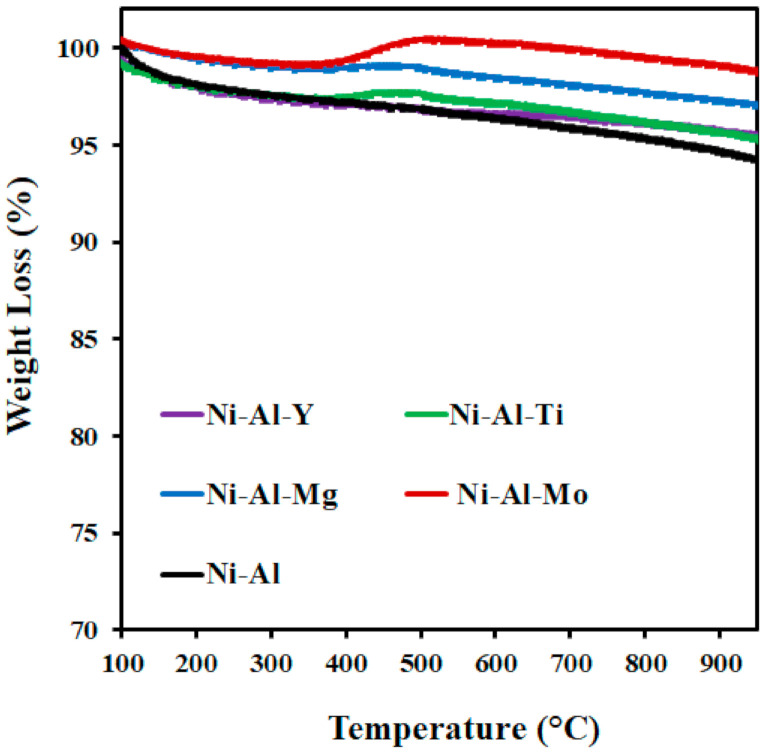
TGA of the spent catalysts tested at 550 °C.

**Figure 6:**
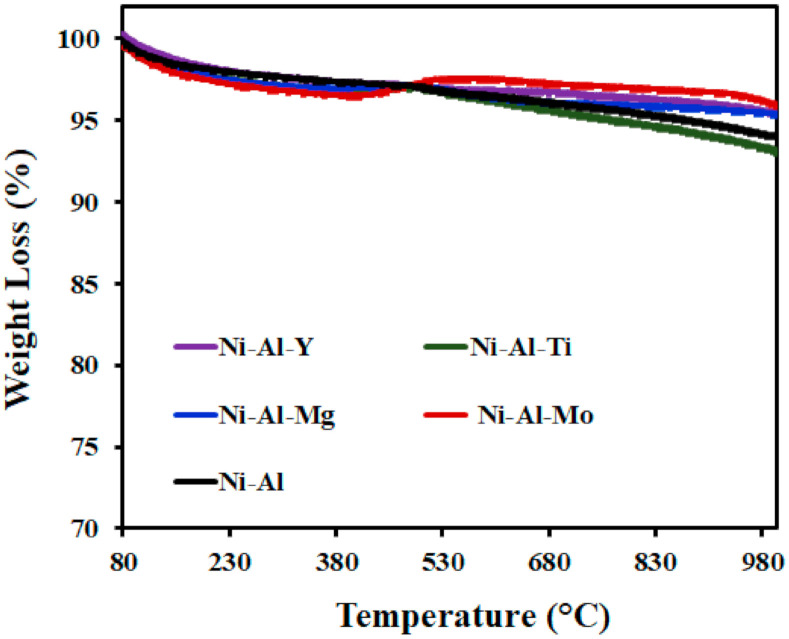
TGA of the spent catalysts studied at 650 °C.

Carbon deposition causes deactivation of the catalyst. TGA technique was employed to investigate the amount of carbon deposits on the used catalysts. The TGA analysis of the spent catalysts tested at 550 °C reaction temperature is given in Figure 5. The quantity of carbon formed on the catalysts was in the range of 1−6 wt.% with the unmodified support having the highest amount and the Mo-modified support having the least amount of carbon deposits. From the TGA profile of each of the catalysts, there was an increase in the weight from 400 to 500 °C. This could be attributed to the oxidization of Ni to NiO in the air atmosphere. The TGA analysis of the spent catalysts studied at 650 °C reaction temperature is given in Figure 6. The quantity of carbon formed on the catalysts was in the range of 4−6.8 wt.% and different for each, while the drop in CH_4_ conversion of the catalysts was 9.3−12.8% in Figure 3, which was related to the experiment time of approximately 8 h. Mo-modified support catalyst had the least amount of carbon deposits while Y-modified support had the highest. For the TGA profile of Mo-modified support catalyst, there was an increase in weight from 430 to 540 °C, which could be attributed to the oxidization of Ni to NiO in the air atmosphere.

Supplementary Figure S4 illustrates the Raman spectra of spent Ni-Al-y (y = 0, Mo, Ti, Y, and Mg) catalysts attained for 7 h using a 550 °C reaction temperature. Two similar intensity peaks appeared at 1473 cm^−1^ and 1535 cm^−1^, corresponding to the D band, ascribed to sp3 hybridized amorphous carbon, and G band, indicated by the occurrence of graphitized carbon, respectively. The D band and G band are characteristic bands of regular-structured carbon that occur on the surface of Ni-Al-y (y = 0, Mo, Ti, Y, and Mg) in the course of the partial reforming reaction. The peak areas ratio of the D and G (I_D_/I_G_) bands is employed to evaluate the graphitic degree and the amount of defects in spent catalysts, a low I_D_/I_G_ ratio denoting higher structural perfection of the spent catalysts. The ratio of I_D_/I_G_ as 1.03, 1.04, 1.10, 1.12 and 1.17 was computed respectively, for Ni-Al-Ti, Ni-Al-Ti, Ni-Al, Ni-Al-Y, Ni-Al-Mo and Ni-Al-Mg catalysts. This displays that the degree of graphitization decreased for spent Ni-Al-Mg, which is consistent with the best catalytic performance of CH_4_ conversion and hydrogen yield. The Raman analysis also depicted that the Ni-Al catalyst possessed the highest peak and highest carbon deposition, while Ni-Al-Mo catalyst had the least, in conformity with the TGA analysis of Figure 5. Supplementary Figure S5 shows the Raman spectra of spent Ni-Al-y (y = 0, Mo, Ti, Y, and Mg) catalysts obtained for 7 h using a 650 °C reaction temperature. Two analogous intensity peaks emerged around 1469 cm^−1^ and 1532 cm^−1^, corresponding to the D band, allocated to sp3 hybridized amorphous carbon, and G band, denoted by the existence of graphitized carbon, respectively. The intensities of the peaks of Ni-Al and Ni-Al-Y were higher than those of the other catalysts. The ratio of I_D_/I_G_ was computed to 0.89, 1.02, 1.03, 1.08, 1.12 and 1.24 for Ni-Al-Y, Ni-Al-Ti, Ni-Al-Mo, Ni-Al and Ni-Al-Mg catalysts, respectively.

## 3. Experimental

### 3.1. Catalyst Development

A wet impregnation procedure was used to obtain the catalysts for the catalyst of 10% Ni supported on 10%X + 80% Al_2_O_3_ (X= Mo, Ti, Y, and Mg). Ninety percent of the support was impregnated with 10% Ni obtained from hydrated nickel nitrate Ni(NO_3_)_2_ × 6H_2_O. The nickel nitrate was first dissolved in 30 mL deionized water followed by the support after having a uniform solution. The catalyst was calcined at 650 °C after drying at 120 °C in a furnace. The prepared catalysts were designated as shown in Table 3.

### 3.2. Catalytic Reaction

A portion of 100 mg of catalyst was used in the partial oxidation of CH_4_ operated atmospherically in a 9.1 mm diameter and 30 cm long tube reactor. PID Eng & Tech Micro provided the reactor. A thermocouple was applied to measure the reaction temperature. The feed gas compositions (methane/oxygen/nitrogen) were in the volume ratio of 3/1.5/2, with a total flow rate of 32.5 mL/min. Reaction temperatures of 550 and 650 °C were used during the experiments. A gas chromatographer (GC-2014 SHIMADZU), connected with a TCD detector using Porapak Q and Molecular Sieve 5A, was used in the analysis of the output gases. CH_4_ conversion and H_2_ yield were calculated using the subsequent equations:

(1) $${\mathrm{CH}}_{4}\;\mathrm{conversion}=\frac{{\mathrm{CH}}_{4,\mathrm{in}}-{\mathrm{CH}}_{4,\mathrm{out}}}{{\mathrm{CH}}_{4,\mathrm{in}}}\;\times 100\;$$

(2) $$H_{2}\;Yield=\frac{moles\;of\;H_{2}\;produced}{2\times {\mathrm{CH}}_{4,\mathrm{feed}}}multiplied\;by\;100$$

### 3.3. Catalyst Description

The new and used catalysts were categorized using the following techniques:

#### 3.3.1. Nitrogen Physical Adsorption

A Micromeritics Tristar II 3020 porosity and surface area analyzer determined the textural properties of the catalysts via adsorption−desorption isotherms using liquid nitrogen. In the test, 0.2–0.3g of the catalyst was used. At first, the sample was heated at 300 °C for three hours to drive away vapor, undesirable adsorbed gases, and organics. The specific surface area of the catalyst was determined via the BET technique and equation.

#### 3.3.2. Temperature Programmed Reduction (TPR)

The H_2_ activation of the catalyst was tested by TPR using an AutoChem-II Micromeritics. In the analysis, 0.070 g of the catalyst precursors were first heated to 150 °C and held at that temperature for 60 min in the presence of Ar at the rate of 1.8 L/h and then cooled to room temperature. Next, the sample temperature was raised to 900 °C at 10 K/min in an automatic furnace at 1 atmosphere. While heating, H_2_/Ar mixture with a volume ratio of 10/90 was flowing at 2.40 L/h. The amount of H_2_ consumed was determined by a thermal conductivity detector.

#### 3.3.3. X-ray Diffractogram (XRD)

The XRD diffraction measurements were performed to detect the crystalline phases of the catalysts. The unit was from XRD Rigaku, having Kα-Cu X-ray radiation of 40 kV and 40 mA, a scanning 2θ range of 10–85° and a step of 0.02°. X’Pert high score plus software was employed to assess the data.

#### 3.3.4. Thermo-Gravimetric Analysis (TGA)

The amount of carbon formation on the surface of the catalyst was examined by thermo-gravimetric analysis through the Shimadzu TGA analyzer. In every analysis, 0.100−0.150 g of used catalyst was heated with a ramping degree of 20 °C/min from ambient temperature to 1000 °C. The mass reduction of the sample as a result of the oxidation in the air was recorded.

#### 3.3.5. Raman Spectroscopy

Raman spectra were performed using an NMR-4500 Laser Raman Spectrometer. A wavelength with an excitation beam of 5.32 × 10^3^ Å was set. A lens with 20× enlargement was employed to assess the spectra. A 6 mW beam power and an exposure time of 3 min were employed. The Raman change of the spectra was calculated in the range 10^3^–3 × 10^3^ cm^−1^. Spectra Manager Ver.2 software was used to manage the profiles.

#### 3.3.6. CO_2_-TPD

The CO_2_ temperature-programmed desorption (TPD) was accomplished via Micromeritics Autochem II apparatus. Initially, 0.006 g of catalyst was activated with helium gas at 600 °C for 1 h and then the sample temperature was reduced to 50 °C. Then CO_2_ was admitted and continued for 60 min. Afterward, He gas was used to flush the sample to take away any physisorbed CO_2_. The peaks of desorption were noted while the temperature was varied by 10 °C/min. The CO_2_ concentration in the output was recorded via a thermal conductivity detector.

## 4. Conclusions

This work has demonstrated the performance effect of alumina support promoters used in synthesizing Ni-based catalysts for methane partial oxidation. The oxide promoters for the support were MgO, MoO_2_, TiO_2_ and Y_2_O_3_. The promoters enhanced the catalytic activity with the exception of the Y_2_O_3_ oxide which inhibited the CH_4_ conversion as well as the H_2_ yield in comparison to the unmodified support. The order of catalytic performance with respect to promoted supports is as follows. For 550 °C reaction temperature: Ni-Al_-_Mg>Ni-Al-Mo>Ni-Al-Ti>Ni-Al-Y and for the reaction at 650 °C; Ni-Al_-_Mg>Ni-Al-Ti>Ni-Al-Mo>Ni-Al-Y. The increase of the reaction temperature from 550 to 650 °C, as expected, improved the catalytic performance and reduced the preference among the type of support promoters. The BET analysis exhibited that the un-promoted catalyst depicted a little bit lower pore volume than the promoted catalysts. From the TPR results, only Ni-A-Mo catalyst showed a peak at lower temperatures, an indication of the existence of free NiO with weak interaction between the promoter and the support. The TGA analysis performed at 550 °C reaction temperature exhibited that Ni-Al catalyst possessed the highest carbon deposit. The Raman analysis displayed amorphous carbon and graphitic carbon deposits.

## Figures and Tables

**Table 1:** Textural properties of Ni-Al-x (x = 0, Mo, Ti, Y, and Mg) catalysts.

Samples	S_BET_ (m^2^/g)	V_P_ (cm^3^/g)	d_p_ (nm)
Ni-Al	173.1	0.613	12.20
Ni-Al-Mo	161.0	0.558	12.42
Ni-Al-Ti	165.0	0.586	12.71
Ni-Al-Mg	172.8	0.603	12.46
Ni-Al-Y	176.3	0.630	12.35

**Table 2:** Assessment of CH_4_ partial oxidation.

Sample	Mass mg	Methane/Oxygen	Space Velocity (ml/min)	Test Temperature (°C)	Methane Conversion (%)	Reference
La Ni_0.5_Nb_0.5_ O_3_	30	2:1	100	750	64	[50]
10% Ni/NiAl_2_O_4_-MgAl_2_O_4_	100	CH_4_: CO_2_: O_2_ 2:1:0.5	140	700	70	[29]
Ni_0.05_Cu_0_._05_Mg_0.9_/Al_0.5_	200	2:1	60	750	88	[1]
5%Ni/Al_2_O_3_	100	2:1	40.6	750	85	[31]
10%Ni+0.1%Rh/Al_2_O_3_	100	2:1	40.6	750	88	[31]
10%Ni+1%Re/γ-Al_2_O_3_	100	2:1	100	600	66.2	[51]
10%Ni/Al_2_O_3_+Mg	100	2:1	32.5	650	92	This work

**Table 3:** Designations of the catalysts used in this analysis.

Sample Name	Sample Formation
Ni-Al	10%Ni/90% Al_2_O_3_
Ni-Al-Mo	10%Ni/10%Mo+80% Al_2_O_3_
Ni-Al-Mg	10%Ni/10%Mg+80% Al_2_O_3_
Ni-Al-Ti	10%Ni/10%Ti+80%Al_2_O_3_
Ni-Al-Y	10%Ni/10%Y+80%Al_2_O_3_

## Supplementary Materials

The following are available online. Figure S1: N_2_ adsorption-desorption isotherms of fresh Ni-Al-x (x = 0, Mo, Ti, Y, and Mg) catalyst. Figure S2: Carbon monoxide yield reaction temperature 550 °C; as a function of time-on-stream over the Ni catalysts. (mass of catalyst, 0.1 g; CH_4_:O_2_ = 2:1, 1 atom; and flow rate, 32.5 mL*/*min.). Figure S3: Carbon monoxide yield reaction temperature 650 °C; as a function of time-on-stream over the Ni catalysts. (mass of catalyst, 0.1 g; CH_4_:O_2_ = 2:1, 1 atom; and flow rate, 32.5 mL*/*min.). Figure S4: Raman spectra of the Ni-Al-y (y = 0, Mo, Ti, Y, and Mg) catalysts obtained at 550 °C reaction temperature. Figure S5: Raman spectra of the Ni-Al-y (y = 0, Mo, Ti, Y, and Mg) catalysts obtained at 650 °C reaction temperature.
