# Supplementary material for: Study of Partial Oxidation of Methane by Ni/Al2O3 Catalyst: Effect of Support Oxides of Mg, Mo, Ti and Y as Promoters
Source: Molecules. 2020 Oct 29;25(21):5029. doi: 10.3390/molecules25215029 (PMC7663497; doi:10.3390/molecules25215029)
Supplement: Supplementary file 1 [file molecules-25-05029-s001.pdf]

molecules

Supplementary Information

Study of Partial Oxidation of Methane by Ni/Al_2O_3 Catalyst: Effect of Support Oxides of Mg, Mo, Ti and Y as Promoters

Ahmed A. Ibrahim [1,*], Wasim U. Khan [1], Fahad Al-Mubaddel [1,2], Ahmed S. Al-Fatesh [1,*], Samsudeen O. Kasim [1], Sofiu L. Mahmud [1], Ateyah A. Al-Zahrani [1], M Rafiq H Siddiqui [3] and Anis H. Fakeeha [1]

1 Chemical Engineering Department, College of Engineering, P.O. Box 800, Riyadh 11421, Saudi Arabia; wasimkhan@gmail.com (W.U.K.); falmubaddel@ksu.edu.sa (F.A.-M.); sofkolajide2@gmail.com (S.O.K.); mahmudsofiu@gmail.com (S.L.M); aazz@ksu.edu.sa; (A.A.A.-Z.); anishf@ksu.edu.sa (A.H.F.)
2 King Abdullah City for Atomic & Renewable Energy: Energy Research & Innovation Center (ERIC) in Riyadh. Riyadh 11451, Saudi Arabia
3 Chemistry Department, King Saud University, P.O. Box 2455, Riyadh 11451, Saudi Arabia; rafiqs@ksu.edu.sa
* Correspondence: aidid@ksu.edu.sa; (A.A.I.); aalfatesh@ksu.edu.sa (A.S.A.-F.)

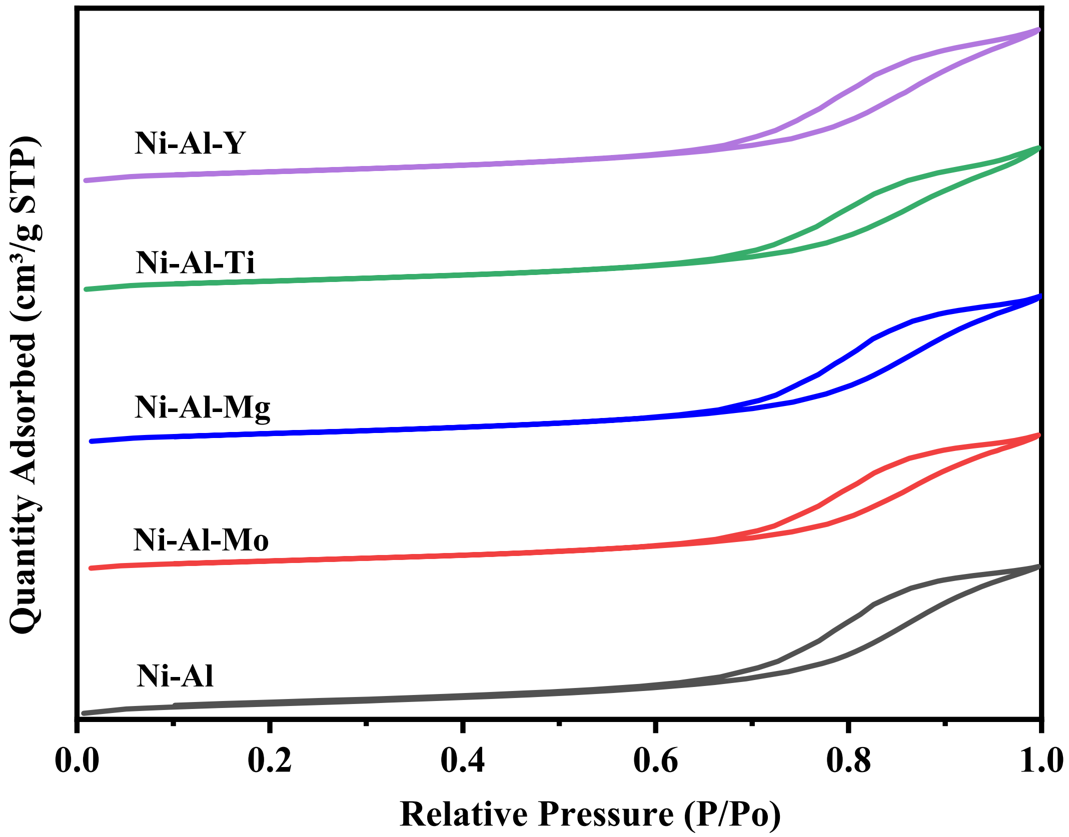

Figure S1. N_2 adsorption-desorption isotherms of fresh Ni-Al-x (x = 0, Mo, Ti, Y, and Mg) catalyst.

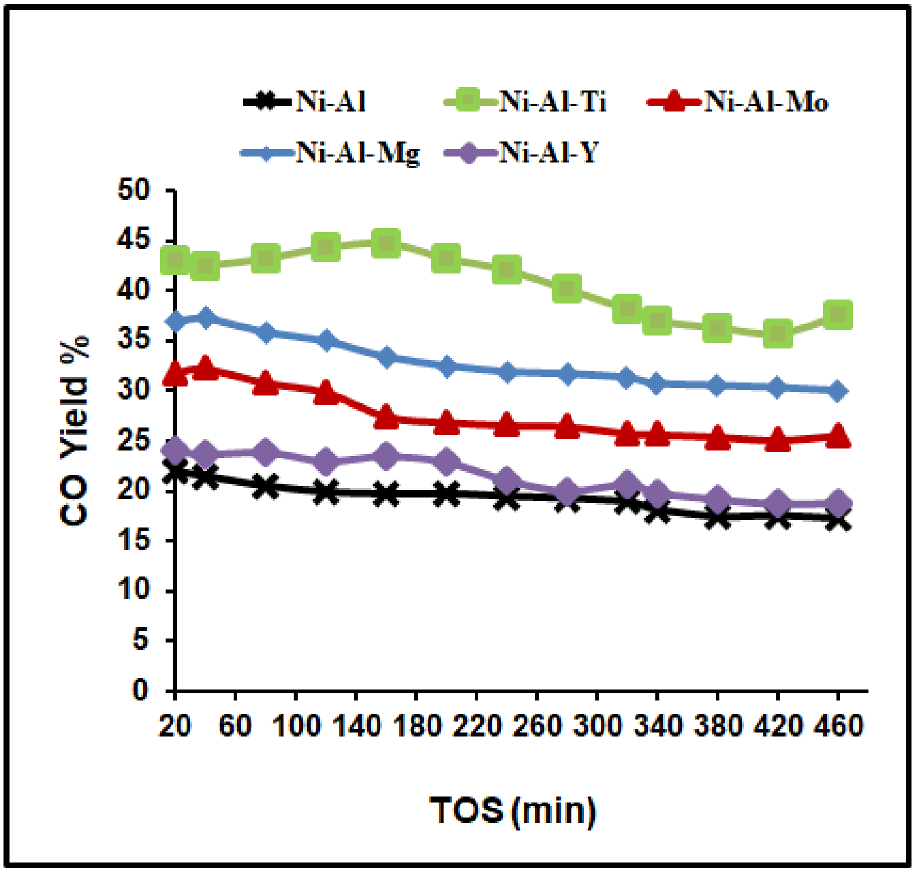

Figure S2. Carbon monoxide yield reaction temperature 550 °C; as a function of time-on-stream over the Ni catalysts. (mass of catalyst, 0.1 g; CH_4:O_2 = 2:1, 1 atom; and flow rate, 32.5 mL/min.).

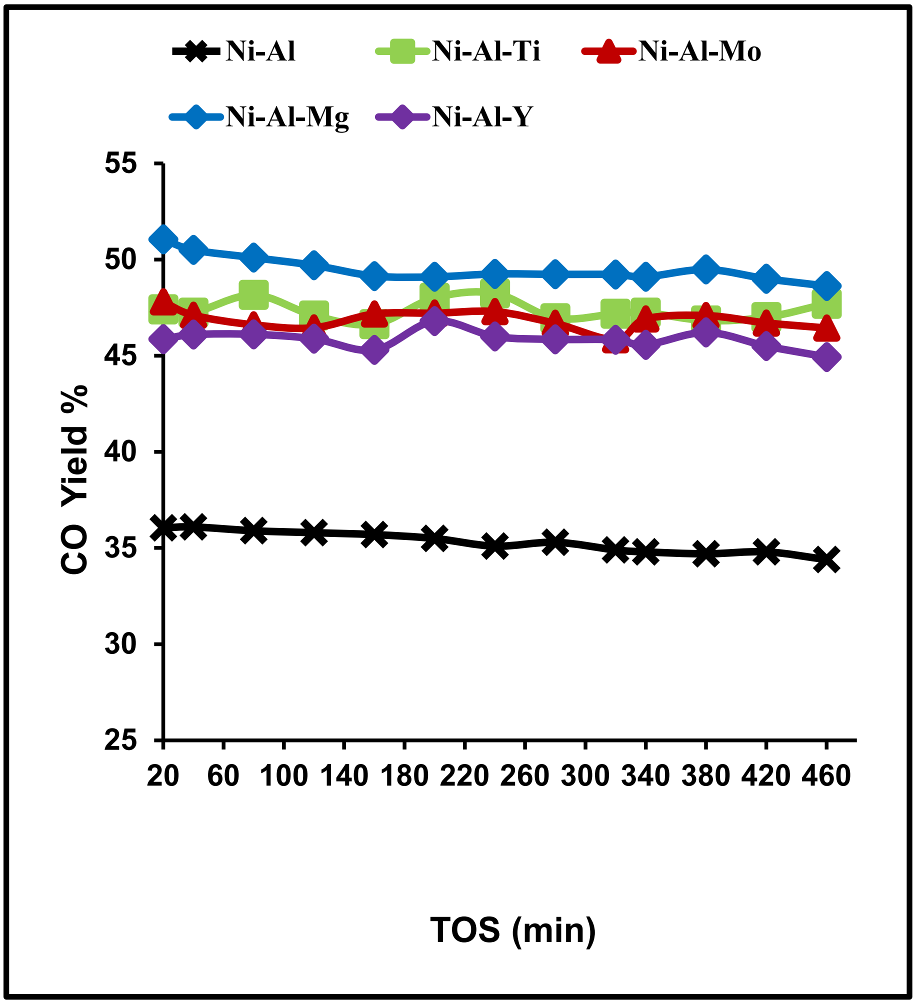

Figure S3. Carbon monoxide yield reaction temperature 650 °C; as a function of time-on-stream over the Ni catalysts. (mass of catalyst, 0.1 g; CH_4:O_2 = 2:1, 1 atom; and flow rate, 32.5 mL/min.).

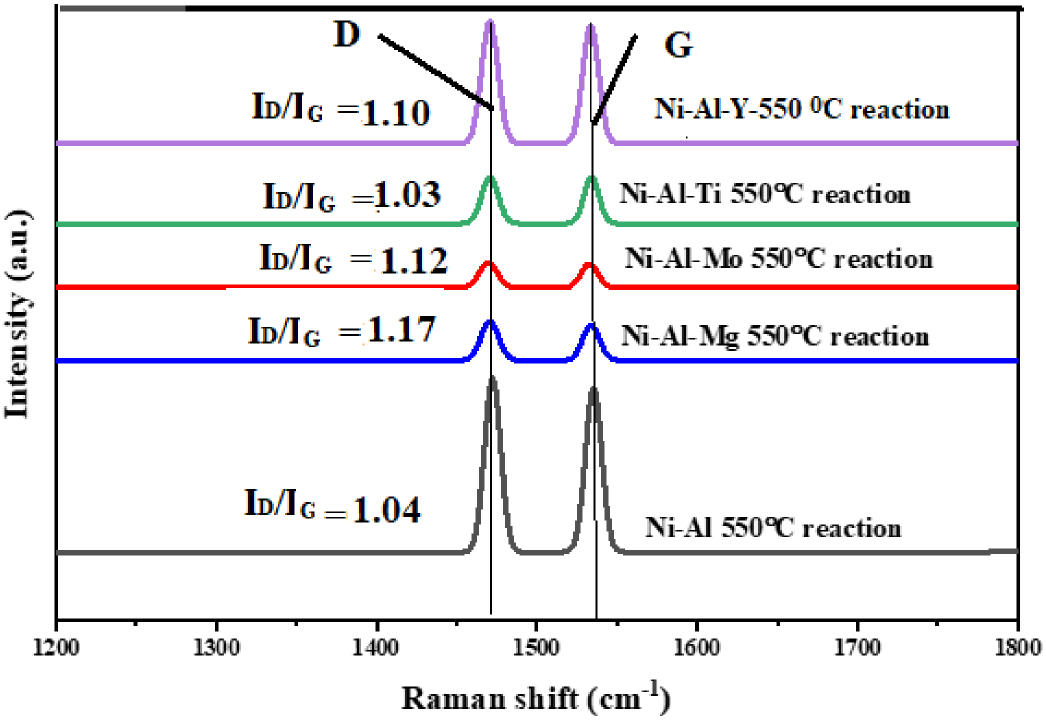

Figure S4. Raman spectra of the Ni-Al-y (y = 0, Mo, Ti, Y, and Mg) catalysts obtained at 550 °C reaction temperature.

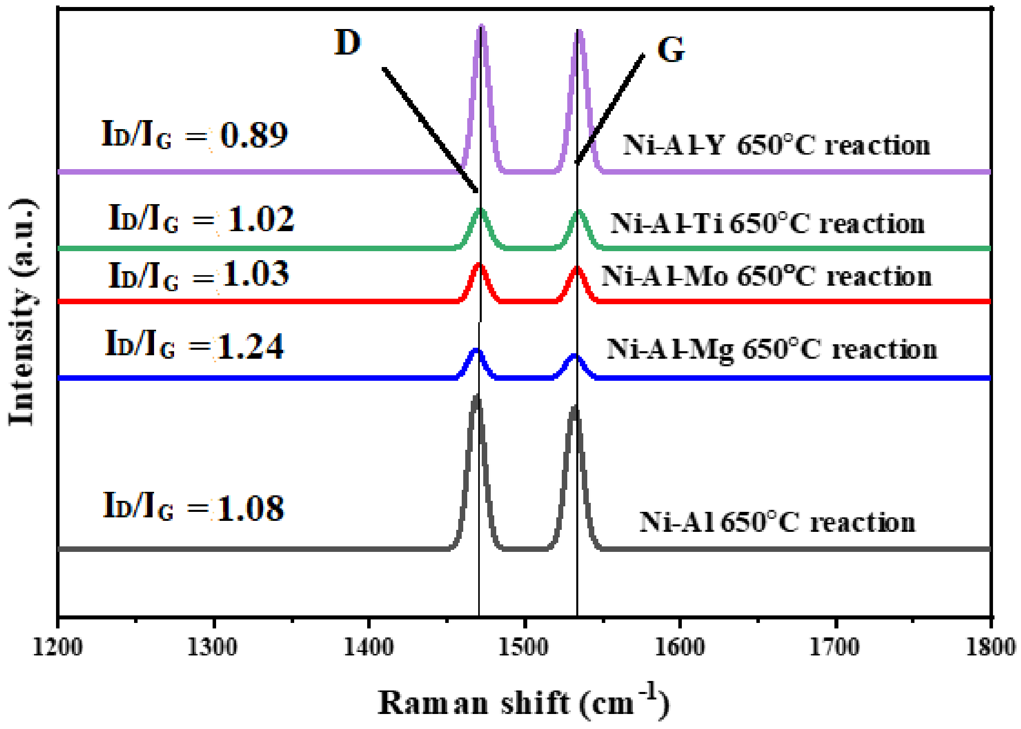

Figure S5. Raman spectra of the Ni-Al-y (y = 0, Mo, Ti, Y, and Mg) catalysts obtained at 650 °C reaction temperature.

Publisher's Note: MDPI stays neutral with regard to jurisdictional claims in published maps and institutional affiliations.

© 2020 by the authors. Licensee MDPI, Basel, Switzerland. This article is an open access article distributed under the terms and conditions of the Creative Commons Attribution (CC BY) license (http://creativecommons.org/licenses/by/4.0/).
